# Implementing Integrated Community-Based Primary Healthcare: Applying the iCoach-Approach to Case Selection to Denmark

**DOI:** 10.5334/ijic.4663

**Published:** 2019-11-04

**Authors:** Stine Bligaard Madsen, Kirsten Beedholm, Flemming Bro, Loni Kraus Ledderer, Louise Ormstrup Vestergård, Viola Burau

**Affiliations:** 1 Central Denmark Region, Viborg, DK; 2 Department of Public Health, Aarhus University, DK; 3 Research Unit for General Practice, Aarhus, DK; 4 Nordregio, Stockholm, SE; 5 Department of Political Science, Aarhus University, DK

**Keywords:** case selection, integrated community-based primary care, implementation, iCoach, Denmark

## Abstract

**Background:**

The iCoach approach to case selection focuses on innovative models of community-based primary healthcare (CBPHC) and their contexts. The aim of this study was to assess the possibilities and limitations of the approach based on Denmark, which differs in significant ways from the jurisdictions initially included.

**Theory and Methods:**

Case study research suggests the approach is an interesting attempt to standardise case selection based on literal replication. The study reviewed the national grey literature and interviewed key informants at national and local levels.

**Results:**

Applying the approach to Denmark required redefining selection criteria related to collaboration and context to capture its specific institutional and policy context. Selecting cases at the organisational level also required assessing how the system level contexts compared to those of the initial three jurisdictions included in iCoach.

**Discussion:**

The iCoach approach allows collecting broadly comparable cases of innovative models of CBPHC across jurisdictions. However, the analysis of underlying conditions of implementing innovative models requires a more interactive approach to case selection.

**Conclusion:**

Researchers need to be clearer about the specific purpose of the case selection. This is also highly relevant for practitioners to ensure that insights are applicable in specific local and national contexts.

## Introduction

Healthcare systems across industrialised countries have turned to integrating health and social services to address common challenges of providing appropriate, equitable and cost-effective care for growing numbers of older adults with complex needs [1,2]. Models of community-based primary healthcare (CBPHC) in particular have proven effective in delivering a range of integrated health and social care services in local settings [3,4,5]. The international research project *Implementing integrated care for older adults with complex health needs* (*iCoach*) has developed an approach to case selection for identifying innovative CBPHC-models and their specific (country) contexts, based on studies of integrated care [1,6]. In many ways, this is similar to the taxonomy of integrated primary care developed by Valentijn and colleagues [7,8]. However, the iCoach approach brings in context already at the stage of the research design and offers a tool kit for the systematic selection of cases that is: innovative models of CBPHM.

From the perspective of the literature on case study research, this is an interesting attempt to adopt a more standardised approach to selecting instrumental cases [9,10]. The iCoach approach does so by defining a set of criteria for innovative CBPHC-models and their contexts, which any potential, new case has to meet. This is an exercise of ensuring comparability between present and new cases as part of a quasi, multiple case design and follows the logic of literal replication [11]. The iCoach approach has emerged in close conjunction with case studies conducted in Ontario, Québec and New Zealand [1,12,13], but applying the criteria to possible cases to a new jurisdiction can raise broader questions about the possibilities and limitations of a more standardised approach to case selection. We set out to maximise variation across jurisdictions and applied the iCoach approach to Denmark, Compared to New Zealand, Ontario and Québec, social care and some primary healthcare services are well integrated administratively at the level of local municipalities as service providers, and Denmark is characterised by a relatively high level of horizontal integration.

## Aim

The aim was to assess the possibilities and limitations of a more standardised approach to case selection based on literal replication using the iCoach approach as an example. We applied the approach to Denmark as a new jurisdiction, which differs in significant ways from the jurisdictions initially included in the iCoach project.

The structure of the article is as follows: in the background we outline the institutional and policy context of community-based primary care in Denmark. The section on theory and methods accounts for how we have adapted the case selection criteria initially developed by iCoach. The results offer an overview of the adapted methods of case selection and the cases selected. The discussion summarises the different ways in which we have applied the iCoach approach and identifies broader implications for future research and practice.

## Background

In the following, we account for the institutional and policy context of community-based primary care in Denmark. This is to identify the central ways in which the Danish healthcare system varies from those jurisdictions, which formed the basis for the iCoach approach to case selection.

### Institutional and policy context of community-based primary care in Denmark

The Danish healthcare system is based on universal access and offers an extensive range of services that are mainly funded through taxation collected at national and local levels. The broader institutional context of the healthcare system is characterised by decentralisation and public corporatism [14]. The national level has overall governing responsibility, while operational responsibility for secondary and primary healthcare is decentralised and lies with the regions and municipalities. Social care services such as care of elderly and people with disability and community mental health services are also highly decentralised and offered by municipalities [15]. National government, regions and municipalities are closely connected through a complex system of negotiations and agreements based on the principle of consensus.

A major administrative reform in 2007 changed the organisational landscape of the Danish healthcare system; it created larger municipalities, replaced 14 counties with five regions and redistributed responsibilities across levels. Today, hospital services are provided by the regions while primary healthcare services are provided by general practitioners (GPs) and by the municipalities [16]. The municipalities also took on rehabilitation, disease prevention and health promotion in addition to their existing responsibilities for health and social care services [17]. The high degree of municipal autonomy in Denmark makes for a relatively strong horizontal integration between social and health care services in administrative terms. In contrast, the increasing specialisation and centralisation of hospital services have highlighted weaknesses in the vertical integration of healthcare services in Denmark, which extends to general practice. Together, this is a distinct characteristic, also compared to the jurisdictions included in the iCoach project.

This is also the main focus of recent policy developments. They include mandatory healthcare agreements between municipalities and regions that outline patient pathways across health and social care services; they are based on a ‘contractual’ form of organisational integration and offer virtual connections across different levels [18]. Another significant development is the extensive prioritisation of health and social services for older people. As part of the national budget agreement in 2014, the parties decided to strengthen elderly care in the municipalities on a long-term basis by setting aside 1 billion DKK (about 134 million EUR) per year [19]. Initially municipalities had to apply for the funds, whereas the funds are now allocated to the municipalities according to a standard distribution formula. In 2016, this was complemented by a national action plan for older people with complex medical needs and the national budget agreements set side 1.2 billion DKK (over 160 million EUR) for the implementation of the plan between 2016 and 2019. The main aim of the plan is to reduce the number of hospital admissions and readmissions and it focuses both on the hospital sector and primary care in the municipalities [16].

Taken together, these policy initiatives have targeted some of the weaknesses in the vertical integration of healthcare services in Denmark and they have supported municipalities to play a more active role in the healthcare system. Still, the dominant focus continues to be hospital-centred; the underlying expectation is that the municipalities close the gap between secondary and primary care by contributing to preventing hospital (re)admissions and decreasing the length of hospital stays especially for older people. This has also shaped the space available for models for integrated care in Denmark, which include follow-up home visits, care coordinators and specific services for patients with chronic diseases. These models are typically either hospital based or disease specific and do not reflect the innovative features of CBPHC models in other countries.

One exception is the municipal intermediate care teams that provide home-based health care primarily to frail elders more broadly. In close collaboration with GPs and/or hospitals, nurses in the teams deliver specialised care such as starting and administering IV antibiotics or fluids; the nurses also deal with less specialised tasks and do so often in close collaboration with other health and social care staff in the municipality [20]. Like CBPHC models in New Zealand, Ontario and Québec these municipal intermediate care teams are organised very differently across municipalities, especially in terms of the collaboration with the GPs and hospitals [20].

From above, the healthcare system in Denmark emerges as decentralised and governed by a form of public corporatism. The horizontal integration of health and social care in the municipalities is relatively strong, whereas the main challenge is the vertical integration between hospitals, GPs and municipalities. This is the focus of recent policy initiatives and municipalities have introduced ‘intermediate care teams’, which can be defined as innovative CBPHC models based on the characteristics identified by Kuluski et al. [12] (collaboration, care provision to geographically defined population, patient-centeredness, care provision to older population). In the following, we discuss how we applied the iCoach approach to case selection in the specific institutional and policy context of Denmark. This will form the basis for identifying individual municipal intermediate care teams as innovative CBPHC in Denmark.

## Theory and Methods

The literature on case study research defines case selection as a process of purposefully identifying individual cases [11,21,22]. The ‘best’ case is the case from which one can learn most in relation to the research question of a given study [9]. This can include intrinsic case studies, where the case itself is of primary interest, and instrumental case studies, which illustrate theoretically based concepts or specific phenomena in particular ways [10]. Instrumental case studies may also include cases from different geographical areas to identify regional, institutional and cultural differences across cases [10,23]. The iCoach approach is concerned with the selection of instrumental cases. Based on a review of the literature on implementing integrated care, the approach defines criteria for innovative models integrating community-based primary care and their specific contexts at different levels. From the perspective of the literature on case study research, this offers a more standardised approach to case selection based on the logic of literal replication. According to Yin [11], the logic is related to the selection of multiple case studies and aims to ensure the ability to predict the same results. The iCoach approach defines criteria for innovative models of integrated CBPHC and their contexts, so that any new cases conform to the very same two sets of dimensions.

This is interesting as the literature typically discusses case selection in relation to the selection of individual or a small number of cases as part of multiple case studies [21,22,24,25,26]. In contrast, the use of predefined selection criteria as part of the iCoach approach offers the possibility of conducting a potentially much larger number of cases by independent groups of researchers, working at different points of time. This may form the foundation for a larger collection of cases from across different jurisdictions. The use of such a collection is highly versatile. It is possible to put individual cases in a broader context to identify similarities and differences rooted in institutional contexts [23,27]. It is also possible to use the cases for new and potentially very different multiple case studies. Applying such a more standardised approach to case selection to a new jurisdiction like Denmark, which varies from the original jurisdictions in significant ways, offers a unique opportunity to assess the possibilities and limitations to standardising case selection based on the logic of literal replication.

### Adapting case selection criteria

The iCoach approach to case selection applied two overall criteria: firstly, that models of CBPHC are innovative; and secondly, that there is maximum variation on relevant contexts among cases. Adapting these criteria to Denmark was important as the application of the adapted criteria formed the basis for identifying innovative intermediate care teams and their specific contexts.

#### Innovative models

Table 1 below gives an overview, how we adapted the specific case selection criteria of innovative models to fit the Danish context.

**Table 1:** Case selection criteria of innovative models.

Original criteria		Adapted criteria

1.	Collaboration (horizontal and vertical)	Vertical integration with hospitals and/or GPs - Effective contact and communication; supported by standardised referral procedures, single contact points or shared assessment instruments - Clear distribution of responsibilities; based on formal agreements or strong informal relations - Mutual trust among providers; for example delegation of specialised medical tasks to intermediate care team
2.	Care provision to geographically defined population	Intermediate care team provides care in municipality only; no blurring of geographical boundaries with collaboration with larger hospitals
3.	Inclusion based on overall assessment of health and social care needs	Intermediate care teams flexible in terms of tasks performed and inclusion not disease specific
4.	Care provision for older people	Exclusion of teams with primary or exclusive focus on patients with mental health problems

A widespread approach to selecting innovative models is to focus on their relative success in terms of specific outcome measures. However, a key lesson underlying the iCoach approach to case selection is that this is difficult because stakeholders attach very different importance to specific outcome measures [12,28]. Our search of grey literature at a national level confirms this and individual evaluations of municipal intermediate care teams in Denmark prioritise outcome measures in very different ways. While some evaluations almost exclusively focus on political goals such as reduced hospital admissions, readmissions and cost effectiveness [29], others are more concerned with user satisfaction and improved care [30]. Evaluations focusing on both types of measures show that intermediate teams generally improve care but do not reduce costs and in some cases may even increase costs [31,32]. Defining success as financial outcome, runs the risk of overlooking intermediate care teams that improve continuity in care and increase user satisfaction. Instead, we followed the pragmatic rationality underlying the iCoach approach and defined innovative models as those models that have the potential to deliver optimal CBPHC to older adults with complex health and social needs. However, we had to adapt the four case selection criteria related to innovative models of CBPHC initially developed by the iCoach approach to fit the specific characteristics of the intermediate care teams in municipalities in Denmark [12].

#### Collaboration in innovative models

The first specific case selection criteria related to municipal intermediate care teams concerns collaboration; horizontally between primary care and home and community care and/or vertical collaboration between primary care with secondary or tertiary providers. We adapted this dimension and focused on vertical integration with hospitals and GPs. This is to reflect the specific institutional context of the healthcare system in Denmark, where services are well integrated horizontally, but where vertical integration between primary and secondary healthcare services continues to be fraught with challenges [33]. Intermediate care teams in Denmark are innovative, when they integrate care across municipalities, GPs and hospitals. Drawing on international studies and national grey literature, we specifically defined vertical integration as follows. First, contact between primary care and secondary care providers should be effective and supported by standardised referral procedures, single contact points or shared assessment instruments [3,5,32,34]. Second, the responsibilities of the primary and secondary care providers, including their collaborative interfaces, should be unambiguous, and preferably included in formal agreements or based on strong informal relations between healthcare providers at the clinical level [3]. Finally, mutual trust among health care providers should be high. Specifically, hospitals and GPs should trust municipal care providers to be qualified to deliver more specialised care and thus delegate medical tasks such as specialised IV antibiotic treatment or fluids in home settings [35].

#### Population of innovative models

The second specific case selection criteria related to municipal intermediate care teams is about care provision to a geographically defined population. It is easy to apply this dimension in a Danish context, where the intermediate teams are municipality-based and therefore naturally only cover the population within this area. There may be some grey zones, where services are strongly integrated with larger hospitals that cover two or more municipalities.

#### Inclusion in innovative models

The third specific case selection criteria related to municipal intermediate care teams concerns person-centredness, that is inclusion based on overall assessment of health and social care needs. This is more distinct in the context of Denmark because some of municipal intermediate teams are disease-specific and have a focus on particular chronic diseases such as diabetes, chronic obstructive pulmonary disease or mental illness [36]. Other teams are more person-centred and target a broader population. Here the emphasis is on being flexible about the tasks performed and including patients based on an assessment of their overall needs rather than any specific needs at the point of access to services [20,37].

#### Target group of innovative models

The fourth and final specific case selection criteria related to municipal intermediate care teams is about care provision for older people. This group typically has complex health and social needs and it is easy to adapt the criteria to Denmark. This group is the key concern of recent policy developments and this has triggered the emergence of a wide range of local initiatives targeting this specific population; there are many local models meeting this particular criterion. However, this excludes a smaller but growing number of models of integration that target primarily or exclusively people with mental illness [38]. Here the same issue of overall vertical integration is at play, but the health and social care providers involved are very different. Inclusion of these models might introduce variation into the study of the implementation, that lies outside the scope of the iCoach approach to case selection.

#### Contexts

Together, these adapted iCoach case selection criteria describe Danish municipal intermediate care teams that have potential to deliver successful CBPHC to older adults with complex needs. The other overall case selection criteria relate to the contexts of municipal intermediate care teams, and cases need to be selected that offer sufficient insights on how different contexts promote or challenge implementation of these models. For an overview of the selection criteria relating to the contexts of innovative models see Table 2 below.

**Table 2:** Case selection criteria of contexts of innovative models.

Original criteria		Adapted criteria

*Organisational level*		
1.	Organizational size	Size of municipality
		- Population size
2.	Resources	Municipal resources
		- Expenditure on health and social care; both absolute and per capita
3.	Geography	Proximity to hospitals and GPs - Distance to closest hospital - Average number of enrolled patients per GP Location of municipality - Urban or rural - Relevant region
*Professional level*		
4.	Prior co-operation among providers	Relative strength of existing co-operation; for example formed through earlier intersectoral projects
5.	Existing multidisciplinary team work	Degree of existing inter-professional teamwork; such as strategic recruitment of specialised nurses, employment of practitioner consultant

We followed the rationale underlying the iCoach approach and prioritised models with maximum context variation located on ‘the margins not the mainstream’ [12]. Since all municipal intermediate teams are part in the same jurisdiction, system level factors such as overall funding, provision and governance of the health and social care system are the same for all innovative models. We therefore focussed on contexts at organisational, professional and clinical levels. Contexts at these levels vary greatly between models and this can be expected to affect the process of implementing the municipal intermediate teams [39]. Our systematic literature search and contact to key stakeholder in municipalities revealed several key contexts and translated this into six case selection criteria.

#### Contexts at organisational level

These contexts of innovative models includes tangible features such as organisational size, resources and geography but also less tangible features such as readiness for change [40,41]. For the purpose of selecting cases, we chose to focus on the former as this was most realistic in light of the time and funding constraints of the scoping study. Further, Evans et al. [40] find that there are close relationships between tangible and non tangible features (e.g. between resources and readiness for change). Maximising variation on tangible features also helps getting at least some variation on intangible features. *Organisational size* was the first specific case selection criteria related to contexts at organisational levels and we defined this as the of innovative models is size of municipality and more specifically population size. *Resources* were the second criteria, which we defined as municipal resources; this included health and social care resources both in absolute terms and per capita. Population size can be expected to affect how strong the demand for implementation of CBPHC is [20], while level of resources can both inhibit or promote implementation of these models [41,42]. Concerning *geography* as the third criteria of the organisational level of contexts, we included two adapted criteria. In the Danish context, the distance between network members and specifically proximity between hospitals and GPs is particularly important. This reflects the specific organisational landscape of the health and social care services, where the vertical integration between healthcare sectors is weak, while the horizontal integration between health and social care in the municipalities is much stronger. We saught to maximise variation based on distance to closest hospital and GP accessibility defined as the average number of enrolled patients per GP in the municipality [43]. This is closely related to other adapted criteria, location of municipality, which can be in either an urban or a rural area. We also selected models across different regions in Denmark. The regions are responsible for hospital services and have specific strategies for developing these services including integration with both municipalities and GPs. These strategies are the result of the negotiations of healthcare agreements at the regional level.

#### Contexts at professional level

Concerning important contexts at this level, both the international and national literature identify the degree of cooperation between inter-organisational network participants prior to implementing healthcare models [37,39,41,42]. Models building on existing relationships tend to have greater success in implementation, although these relationships may also hinder implementation of individual elements of the model [39]. We used prior co-operation among providers as the fourth specific case selection criteria and aimed for maximum variation to understand how these local relationships affect implementation. Another context at the professional level was the degree of inter-professional teamwork within the municipalities. Some municipal intermediate care teams in Denmark are multidisciplinary while others are mono-professional [20,37]. Existing multi-disciplinary team work was the fifth specific case selection criteria and variation was expected to affect implementation in terms of how the models are framed to match professional interests.

International studies say less about contexts at the clinical level. The search of grey literature at the national level also offered no real insights into which contexts might be relevant at this level. We therefore chose to maximise variation in terms of the other contexts we identified at the organisational and professional levels. However, this does not mean contexts at the clinical level do not have any effect, but we did not have any knowledge about potential effects prior to our analysis and we could not select cases based on contexts at the clinical level.

## Results

### Adapting methods of case selection

Based on the adapted case selection criteria, the next step was to identify local models of municipal intermediate care teams meeting the criteria. We began by using traditional methods of case selection and searched databases. We then widened our search to include grey literature. However, we only found a small number of local models that had been evaluated; the evaluations also focused on issues of the design and the outcomes of the local models, whereas the concern for context remained largely implicit [30,31,37]. Further, these models represented typical cases with low potential for identifying rich context data.

We therefore followed the more pragmatic methods of case selection developed as part of the iCoach approach. This meant drawing primarily on knowledge and expertise from key stakeholders to identify models of CBPHC ‘on the margins and not the mainstream’ [12]. Specifically we contacted both government agencies and relevant interest organisations with comprehensive knowledge of integrated networks in community based primary care. These included the Board of Health (*Sundhedsstyrelsen*) and the Ministry of Health at government level as well as the Association of Municipalities (Kommunernes Landsforening) and Regions (*Danske Regioner*), the Professional Association of General Practitioners (*Praktiserende Lægerns Organisation*) and Nurses (*Dansk Sygepleje Råd*) as interest groups. The public corporatist nature of the Danish healthcare system gives these actors key roles in health policy and governance, and as informal sources they gave us valuable knowledge of diverse or even extreme cases, which were not identified in the available literature. Our informants also pointed to other potential informants with knowledge of specific municipal intermediate care teams.

Following the importance of informal sources highlighted in the iCoach approach [12], we used this snowballing method to identify potentially interesting models for case selection. We explored individual local models by conducting information interviews with local informants and collecting additional information from official documents. We used this as a basis for writing short case descriptions to account for the individual innovative models and their contexts according to the specific case selection criteria outlined in the previous section. This resulted in a list of five local models that were easily comparable and that offered an informed basis for the final case selection.

### Selecting cases for implementation study

Applying the adapted case selection criteria and methods resulted in the selection of three intermediate care teams located in the municipalities of Aarhus, Slagelse and Varde respectively; the underlying rationale was to maximise variation in terms of models and contexts. The three cases all fulfil the criteria for innovative models of CBPHC but vary greatly in terms of the organisation of vertical integration and the contexts the models are embedded in.

#### Innovative models

Table 3 gives on overview of the cases based on the case selection criteria related to innovative models.

**Table 3:** Selection criteria of municipal intermediate care teams – innovative models.

AARHUS	SLAGELSE	VARDE
Hospital-based model of integration	Institutionally supported GP-based model of integration	Informally supported GP-based model of integration

Strong integration with hospital geriatric team	Strong integration with GPs	Strong integration with GPs
Standardised referral procedures, single point of contact, shared assessment instruments Formal agreements on distribution of responsibility; relatively high mutual trust (core tasks)	Standardised referral procedures, single point of contact, shared assessment instruments No formal agreements, but strong informal relations supported by municipal GP; high mutual trust (extensive delegation of medical tasks)	Standardised referral procedures, single point of contact No formal agreements, informal relations informally supported over long period of time; high mutual trust (extensive delegation of medical tasks)

Care provision for people living in municipality	Care provision for people living in municipality	Care provision for people living in municipality

Focus on complex needs, may include more or less specialised health and social care services	Focus on acute care needs and complex health and social care needs (outside existing municipal health and social care system)	Focus on acute care needs and needs for specialised health care

Frail elders with comorbidity as primary target group	Elderly people with complex needs as main target group	Elderly people and people with comorbidity as principal target group

#### Vertical integration in innovative models

All three cases are characterised by strong vertical integration with hospitals or GPs. The focus and organisation of the integrational ties with either hospitals or GPs vary greatly among the municipal intermediate care teams in Denmark. This reflects the general challenges associated with vertical service integration together with the highly decentralised nature of the health and social care system in Denmark. In Aarhus, the local model of integration is hospital based and the intermediate care team has strong ties with the hospital based geriatric team; this includes standardised referral procedures, a single point of contact and shared assessment instruments. Formal agreements on the distribution of responsibilities and relatively high levels of mutual trust further support integration. The integration with GP services is more moderate with no formal agreements in place and uneven levels of trust between the intermediate care teams and the GPs. Conversely, the local models of integration in Slagelse and Varde are GP-based and the intermediate care teams have stronger ties with GP practices. This includes standardised referral procedures and a single point of contact in both cases and additionally use of shared assessment instruments in the case of the Slagelse. There are no formal agreements, but strong informal relations ensure a clear distribution of responsibilities and this is supported in different ways. In Slagelse support is institutionalised in form of a GP employed by the municipalities, whereas the support in Varde is informal and rooted in a history of close cross-sectoral ties. Further, both cases have extensive delegation of medical tasks from GPs to the nurses in the intermediate teams, which indicates high levels of mutual trust.

#### Population of innovative models

The three intermediate care teams only provide services to people living in the respective municipality and thus fulfil the case selection criteria care provision to geographically defined population.

#### Inclusion in innovative models

The same applies, to the selection criteria inclusion based on overall assessment of health and social care need. The teams have an overall focus on more acute and complex needs and remain flexible in terms of the particular services delivered, while the specific inclusion criteria differ somewhat.

#### Target group of innovative models

Care provision for older people is the main concern of all three teams. The teams in both Aarhus and Varde also provide home care to other groups such as cancer patients.

In summary, selected intermediate care teams in Aarhus, Slagelse and Varde meet the adapted criteria for case selection related to innovative models of CBPHC. There is some variation of local models of integration based on either hospitals or GPs, but all three cases have the potential for delivering optimal care to older adults with complex needs. This offers a strong basis for studying the implementation of models of CBPHC in a Danish setting.

#### Context

As Table 4 below shows, the intermediate care teams are part of three very different municipalities and this offers a solid basis for analysing how contexts at organisational and professional levels affect implementation.

**Table 4:** Selection criteria of municipal intermediate care teams – contexts of innovative models.

Case selection criteria		AARHUS	SLAGELSE	VARDE

		Hospital-based model of integration	Institutionally supported GP-based model of integration	Informally supported GP-based model of integration
*Organisational level*				
1.	Size of municipality	Second largest municipality in Denmark; population of 340 421^1^	One of larger municipalities in Denmark; population of 78 968^1^	One of smallest municipality in Denmark; population of 50 301^1^
2.	Municipal resources	1 410 981 000 DKK^2^	398 750 000 DKK^2^	244 780 000 DKK^2^
3.	Proximity to hospitals and GPs	High for both hospital and GPs Largest hospital in Denmark located in municipality Average number of patient pr. GP less than 1,600; less than one third of GPs older than 60	High for hospital, medium for GPs Smaller hospital located in municipality Average number of patient pr. GP between 1,600 and 1699; up to 39 per cent GPs older than 60	Low for hospital, medium for GPs Nearest hospital located in 30–50 km radius Average number of patient pr. GP less than 1,600; more than one third of GPs older than 60; long distance in more rural areas as geographically large municipality
4.	Location of municipality	Mainly urban areas Part of Central Denmark Region	Urban and rural areas Part of Region Sealand	Large rural areas Part of Southern Denmark Region
*Professional level*				
5.	Prior co-operation among providers	Strong ties Many cross sectoral projects between municipal healthcare providers and geriatric hospital ward	Selected, fairly strong ties Well-functioning follow-up home visits between municipal healthcare providers and some GP practices	Individual, strong ties No specific programme for building ties, but do exist in relation to individual care trajectories
6.	Existing multidisciplinary team work	Team of specialist nurses with 5+ years experience, nurses and nurse assistants	Team of specialist nurses with relevant experience, nurses and nurse assistants; municipal GP offers further liaising	Team of specialist nurses with relevant experience, nurses and nurse assistants

*Notes*: ^1^ Figures from 2017; Statistics Denmark, StatBank Denmark: FOLKA: FOLK1A: POPULATION AT THE FIRST DAY OF THE QUARTER BY REGION, SEX, AGE AND MARITAL STATUS. (http://www.statistikbanken.dk/statbank5a/SelectVarVal/Define.asp?MainTable=FOLK1A&PLanguage=1&PXSId=0&wsid=cftree). ^2^ Figures from 2017; Statistics Denmark, StatBank Denmark: REGK11: MUNICIPALITY ACCOUNTS (DKK 1,000) BY REGION, MAIN ACCOUNT, DRANST AND KIND. (http://www.statistikbanken.dk/10188).

#### Organisational level of contexts

Firstly, the size of municipality varies greatly; Aarhus is the second largest municipality in Denmark and Slagelse is one of the larger ones, while Varde is one the smallest municipalities in the country. In broad terms, and secondly, this is reflected in municipal resources. Thirdly, there are also interesting differences in the proximity to hospitals and GPs. This is high all around for Aarhus, which has the country’s largest hospital and where the number of patients per GP and the age of GPs is below average. Proximity is lower in the other two cases with medium proximity to GPs and high to low proximity to hospitals. Slagelse has a smaller hospital, whereas the nearest hospital in Varde is located 30–50 km away. These variations, fourthly, are also related to the location of municipality. Aarhus covers mainly urban areas, Slagelse a mix of urban and rural areas, while Varde emcompasses large rural areas. The three municipalities are also part of three different regions.

#### Professional level of context

Fifthly, there are different patterns of prior co-operation among providers. There are strong ties in Aarhus that have evolved from many cross sectoral projects between municipal health care providers and the geriatric hospital ward. In Slagelse, there are fairly strong ties with selected GP practices based on a well-functioning programme of follow-up home visits. The picture is similar in Varde, where strong ties with GPs exist but only in relation to individual care trajectories. Finally and sixthly, across the three municipalities existing multidisciplinary team work builds on a combination of specialist nurses, nurses and nurse assistants. In Slagelse, a GP employed full time by the municipality supports the team with additional liaising.

## Discussion

The iCoach approach to case selection aims to identify innovative models of CBPHC and their specific contexts to support implementation and scaling-up. From the perspective of the literature on case study research, this is an interesting attempt to adopt a more standardised approach to selecting cases based on the logic of literal replication. The iCoach approach has emerged in close conjunction with (country-) specific case studies and the aim of the paper was to assess the possibilities and limitations of standardising case selection by applying the approach to a new jurisdiction. We thus maximised variation across jurisdictions by selecting Denmark, which is characterised by a distinct combination of strong horizontal but weak vertical integration.

The distinct characteristics of the institutional and policy context of the health care system in Denmark had significant ramifications for applying the predefined selection criteria of the iCoach approach. This required redefining the first criteria of collaboration and adding sub-criteria to take account of the particular characteristics of municipal intermediate care teams and the ways in which they vertically integrated with hospitals and/or GPs. The redefinition was also necessary, as the other selection criteria applied equally across all intermediate care teams and did not offer any lever to discriminate among individual teams as possible cases. We also had to redefine the case selection criteria related to the contexts of innovative models. The iCoach approach initially identified contexts at four levels, whereas we included only two. The political-administrative level is less relevant in Denmark; the country has a unitary state and the local models of intermediate care teams thus belong to the same jurisdiction. Based on the international and national literature it was also difficult to specify relevant variations of contexts at the level of practice that could be formulated as case selection criteria. Concerning the two remaining levels of contexts, we also here developed specific definitions that reflected the peculiarities of the healthcare system in Denmark. We focused on the tangible dimensions of contexts at the organisational level and included four case selection criteria, while we captured contexts at the professional level through two specific case selection criteria.

Applying the iCoach approach to case selection to Danish CBPHC involved much more than merely adapting the predefined selection criteria for innovative models of CBPHC and their contexts; it required redefining several selection criteria to capture the distinct characteristics of the institutional and policy context of the health care system in Denmark. This points to some limitations of a more standardised approach to selecting cases based on the logic of literal replication, which we discuss in more detail below.

The process of redefining the initial selection criteria of the iCoach approach also revealed a second level of case selection. Selecting cases at the organisational level in the new jurisdiction, required assessing how the institutional and policy contexts of the health care system in Denmark compared to those of the initial three jurisdictions included in the iCoach project. This turned out to be an exercise of indirect case selection at the systems level. A distinct feature of the iCoach approach is that the criteria for case selection include but go beyond the system level, reflecting a ‘whole systems’ approach [44]. This is otherwise the typical level for case selections in cross-country comparative research designs [45]. In this way, the case selection of innovative models of CBPHM and their contexts builds on coherence between the different dimensions of the approach (the model on the one hand and the context on the other). Our analysis demonstrates that there is a tension between coherence within the individual country case and coherence across country cases. This is a salient tension in case study research between the focus on the individual case on the one hand and the comparative approach, which looks across cases [22,46].

The iCoach approach demonstrates a commitment to taking context seriously and to bringing it in early on, already as part of the process of case selection. This is important because context is highly relevant for understanding local differences in implementation processes [27]. However, our analysis revealed that there are some limits to boxing in context by applying a more standardised approach to selecting multiple cases based on predefined criteria. Overall, the iCoach approach travelled well; the individual dimensions of innovative models and their contexts were broad enough to have clear resonance with the case of Denmark. However, identifying the underlying, specific conditions for the implementation of the particular, local models of innovative intermediary care teams required detailed redefinitions of individual dimensions.

The possibilities and limitations of the iCoach approach as an example of a more standardised approach to case selection reflect a trade-off between the general and the particular, which is salient in case study research [9,10]. The iCoach approach offers the possibility of collecting broadly comparable cases of innovative models of CBPHC across different jurisdictions. The iCoach approach further underpins this: by defining innovative models in terms of their potential to deliver optimal care; by identifying such models especially based on informal sources; and by selecting cases from a range of contexts within individual jurisdictions [1,12,27]. In contrast, the analysis of underlying, specific conditions of implementing individual innovative models of CBPHC, requires a more interactive approach to selecting either a single or multiple cases. This is in line with Yin’s [11] observation that literal replication builds on the results individual cases produce and how these results compare to those of other cases. This always also includes the possibility that individual cases challenge the initial theoretical propositions and require a revision of selection criteria. These feedback loops are crucial for producing context-specific insights into the implementation of innovative models of CBPHC. This ensures that insights have high relevance for managers and professionals working with strengthening the integration of CBPHC.

## Conclusion

Our study has important implications for future research and practice related to identifying innovative models of CBHC and their contexts. When researchers apply the iCoach approach and similar approaches offering more standardised case selection based on literal replication, they need to be clearer about the specific purpose of the case selection. Is this about identifying broadly comparable, innovative models of CBPHC? Or is this about identifying specific, underlying conditions for implementing innovative models? This distinction is also highly relevant from a practice perspective. The feedback loops built into a more interactive approach to case selection ensure applicability. This means that the insights into the implementation of innovative models of CBPHC are sensitive to local and national contexts.
